# Urinary retinol binding protein predicts renal outcome in systemic immunoglobulin light‐chain (AL) amyloidosis

**DOI:** 10.1111/bjh.17706

**Published:** 2021-08-09

**Authors:** Tamer Rezk, Rashim Salota, Jaslyn J. Gan, Helen J. Lachmann, Marianna Fontana, Keith Siew, Ana Martinez‐Naharro, Christianne Guillotte, Paul Bass, Sajitha Sachchithanantham, Shameem Mahmood, Aviva Petrie, Carol J. Whelan, Jennifer H. Pinney, Mark Dockrell, Darren Foard, Thirusha Lane, Ashutosh D. Wechalekar, Philip N. Hawkins, Stephen B. Walsh, Julian D. Gillmore

**Affiliations:** ^1^ Division of Medicine National Amyloidosis Centre University College London London UK; ^2^ Division of Medicine UCL Department of Nephrology University College London London UK; ^3^ Epsom and St Helier’s University Hospitals London UK; ^4^ UCL Eastman Dental Institute London UK

**Keywords:** amyloid, myeloma, chemotherapy, renal medicine

## Abstract

Renal risk stratification in systemic immunoglobulin light‐chain (AL) amyloidosis is according to estimated glomerular filtration rate (eGFR) and urinary protein creatinine ratio (uPCR), the latter attributed to glomerular dysfunction, with proximal tubular dysfunction (PTD) little studied. Urinary retinol binding protein 4 (uRBP), a low molecular weight tubular protein and highly sensitive marker of PTD, was prospectively measured in 285 newly diagnosed, untreated patients with systemic AL amyloidosis between August 2017 to August 2018. At diagnosis, the uRBP/creatinine ratio (uRBPCR) correlated with serum creatinine (*r* = 0·618, *P* < 0·0001), uPCR (*r* = 0·422, *P* < 0·0001) as well as both fractional excretion of phosphate and urate (*r* = 0·563, *P* < 0·0001). Log uRBPCR at diagnosis was a strong independent predictor of end‐stage renal disease {hazard ratio [HR] 2·65, [95% confidence interval (CI) 1·06–6·64]; *P* = 0·038}, particularly in patients with an eGFR >30 ml/min/1.73 m^2^ [HR 4·11, (95% CI 1·45–11·65); *P* = 0·008] and those who failed to achieve a deep haematological response to chemotherapy within 3 months of diagnosis [HR 6·72, (95% CI 1·83–24·74); *P* = 0·004], and also predicted renal progression [HR 1·91, (95% CI 1·18–3·07); *P* = 0·008]. Elevated uRBPCR indicates PTD and predicts renal outcomes independently of eGFR, uPCR and clonal response in systemic AL amyloidosis. The role of uRBPCR as a novel prognostic biomarker merits further study, particularly in monoclonal gammopathies of renal significance.

## Introduction

The amyloidoses are disorders of protein folding, in which a variety of proteins misfold and aggregate into fibrils that accumulate in tissues and disrupt organ function.^1^ Immunoglobulin light‐chain (AL) amyloidosis is the most common and serious type of systemic amyloidosis.^2^ Renal and cardiac involvement are each present in ~70% of patients at diagnosis manifesting with proteinuric renal impairment ^3^ and congestive cardiac failure respectively.

The three key biomarkers known to predict renal outcomes in AL amyloidosis are serum albumin, proteinuria and estimated glomerular filtration rate (eGFR). Current risk stratification is based upon ‘Renal Staging’ at diagnosis with the combination of proteinuria >5 g/24 h and eGFR <50 ml/min/1·73 m^2^ predicting progression to dialysis.^4^ Clonal response to chemotherapy is also a strong determinant of both patient and renal survival.^5^ However, a proportion of patients with renal AL amyloidosis who have proteinuria of <5 g/24 h at diagnosis progress to end‐stage renal disease (ESRD) and conversely, many patients with proteinuria of >5 g/24 h do not progress, highlighting the limitations of risk stratification using 24‐h proteinuria and eGFR alone. Proximal tubular dysfunction (PTD) has largely been ignored in favour of glomerular dysfunction in AL amyloidosis, and its significance is poorly understood.

Presence in the urine of retinol binding protein 4 (uRBP), a low molecular weight protein that is filtered at the glomerulus and almost completely reabsorbed via the proximal tubule,^6^, ^7^, ^8^ is a well‐recognised biomarker of PTD in diseases characterised by the renal Fanconi syndrome such as those associated with plasma cell dyscrasias.^9^ Urinary RBP has been shown to be an early indicator of renal tubular injury in patients with multiple myeloma and may be a more sensitive marker than serum creatinine for detecting renal dysfunction in this cohort.^10^

We undertook a prospective study of the uRBP/creatinine ratio (uRBPCR) in newly diagnosed, untreated patients with systemic AL amyloidosis referred to the UK National Amyloidosis Centre to determine the presence, extent and effect on renal outcomes of PTD and its correlation with histopathological examination.

## Patients and Methods

### Patients

A total of 285 treatment‐naïve patients who attended the National Amyloidosis Centre with newly diagnosed systemic AL amyloidosis between August 2017 and August 2018 were enrolled into a prospective study in which uRBP was measured in conjunction with routine clinical, biochemical, echocardiographic and scintigraphic assessments, conducted according to the AL Chemotherapy Study (ALchemy) protocol.

Renal involvement by amyloid at study entry (baseline) was defined as non‐Bence Jones proteinuria of >0. 5 g/24 h and cardiac involvement was defined by echocardiography, according to International Amyloidosis Consensus Criteria,^11^ or with additional cardiac magnetic resonance imaging, as appropriate.^12^, ^13^

All patients underwent haematological assessments every 1–3 months at the National Amyloidosis Centre, comprising serum free light chain (FLC) assay, serum and urine immunofixation electrophoresis as well as clinical evaluation, serum and urine biochemistry, echocardiography and serum amyloid P component (SAP) scintigraphy ^14^ every 6 months.

All patients were managed in accordance with the Declaration of Helsinki and provided written informed consent for study entry (REC reference: 09/H0715/58) and publication of their data.

### Quantification of proteinuria

The urinary protein/creatinine ratio (uPCR) and urinary albumin/creatinine ratio (uACR) were measured using standard biochemical assays. The uRBP was measured using a manual two‐site two‐step sandwich enzyme‐linked immunosorbent assay (ELISA), as previously described,^15^ and results were expressed as the uRBPCR in µg/mmol, as previously described.^16^

### Renal Histology

Renal biopsies were performed in 119 of 285 patients. All biopsies were routinely stained with Congo red and a panel of amyloid‐fibril antibodies, as previously described.^17^ Additionally, all biopsies containing sufficient cortical tissue for evaluation (104/119) were analysed by a renal histopathologist, blinded to the study results, and assigned an ‘Index of Chronic Damage’ category of T0 (<25%), T1 (26–50%) or T2 (>50%) according to the previously described Modified Oxford Score.^18^

### Renal outcomes

Renal survival was defined by a requirement for renal replacement therapy (ESRD). Renal progression at 6 months was defined as a ≥25% loss of eGFR from baseline and/or >30% increase in proteinuria^4^ and renal progression at 12 months by a ≥25% loss of eGFR from baseline and/or >50% increase in proteinuria.^11^ Patients who presented with ESRD (*n* = 2) were excluded from all analyses of renal survival and renal progression.

### Response to chemotherapy

Haematological response to chemotherapy was evaluated at both 3 and 6 months from baseline and defined according to previously validated criteria. Briefly, patients were stratified into complete (CR) or very good partial (VGPR) haematological responders and compared with partial (PR) and non (NR) haematological responders.^3^, ^5^

### Statistical analysis

Normally distributed variables were presented as mean (range) and non‐normally distributed variables as median [interquartile range (IQR)]. Pearson’s correlation coefficients were estimated between Log uRBPCR and other variables of interest. Patients in whom data points were missing at set time‐points were excluded from relevant analyses. Variables of interest such as eGFR and uPCR were analysed as both continuous and categorical variables. Multivariable linear regression was used to analyse the relationship between Log uRBPCR and those variables found to be statistically significant at the 10% level in univariable linear regression. Cox proportional hazard regression analysis was used to investigate the variables independently associated with either death, dialysis (renal survival) or renal progression at 6 and 12 months after study entry. Given the independent prognostic value of baseline uRBPCR on renal outcomes, the optimal uRBPCR value predicting renal survival was sought by receiver operator characteristic (ROC) curve analysis and found to be 116 µg/mmol (sensitivity of 93% and specificity of 66%). Consequently and for clinical ease, patients were categorised into those with a baseline uRBPCR of ≥100 or <100 µg/mmol for analysis of renal survival by Kaplan–Meier estimate.

To avoid spuriously significant results arising from multiple testing, a significance level of 0·01 was used unless otherwise specified. Statistical analyses were performed using the Statistical Package for the Social Sciences (SPSS®) version 25·0 (IBM SPSS Statistics for Windows released 2017. IBM Corp., Armonk, NY, USA) and Stata release 15 (Stata Statistical Software 2017. StataCorp., College Station, TX, USA).

## Results

### Baseline demographics

Baseline demographics of all 285 patients are listed in Table I. The median (range) uRBP was 231·0 (9·9–31 768) µg and the uRBPCR was 37·6 (2·45–11 832) µg/mmol. Values at study entry for serum creatinine, eGFR [Modification of Diet in Renal Disease (MDRD) formula], measured creatinine clearance (ml/min/1 73 m^2^), serum albumin, 24‐h urinary protein excretion, uPCR, uACR, N‐terminal pro‐B natriuretic peptide (NT‐proBNP), Troponin T (TnT), kappa and lambda serum free light chains (sFLC), as well as details on chemotherapy regimens are shown in Table I. Other features of PTD including increased fractional excretion of phosphate (FE_PO4_ >20%) and urate (FEx >10%) were present in 73/285 (26%) and 39/285 (14%) patients respectively. Among 104 patients who had evaluable renal histology, 65 (63%) had a mild degree of chronic damage (T0), 14 (13%) a moderate degree of chronic damage (T1) and 25 (24%) had severe chronic damage (T2).

**Table I:** Baseline demographics of all patients.

Demographic or clinical characteristic	Value
Total number of patients	285
Age years, median (range)	70 (30–93)
Patients with renal involvement, *n* (%)	208 (73)
Patients with cardiac involvement, *n* (%)	182 (64)
Serum creatinine, mmol/l, median (range)	94 (34–609)
eGFR, ml/min/1 73 m^2^, median (range)	66 (10–100)
CKD Stage, *n* (%)	
1	56 (20)
2	105 (37)
3A	41 (14)
3B	32 (11)
4	38 (13)
5	13 (5)
CrCl, ml/min, median (range)	68 (4–274)
Serum albumin, g/l, median (range)	34 (13–53)
24‐h urinary protein loss, g, median (range)	2·8 (0·1–42)
Urinary protein creatinine ratio, mg/mmol, median (range)	334 (8–5045)
Urinary albumin creatinine ratio, mg/mmol, median (range)	147 (1–>4000)
NT‐proBNP, ng/l, median (range)	2111 (<50–204 222)
Troponin T, ng/l, median (range)	45 (2–458)
Amyloidogenic light chain, *n* (%)	
Lambda	219 (77)
Kappa	66 (23)
Bence Jones protein, *n* (%)	
Present	197 (70)
Absent	88 (30)
λ sFLC in AL (lambda) amyloid (*n* = 219), mg/l, median (range)	174 (13–2865)
κ sFLC in AL (kappa) amyloid (*n* = 66), mg/l, median (range)	301 (13–3130)
Index of Chronic damage on renal histology, *n* (%)	104
T0 (mild)	65 (63)
T1 (moderate)	14 (13)
T2 (severe)	25 (24)
Chemotherapy regimen, *n* (%)	
Velcade	245 (86)
Rituximab	11 (4)
Immunomodulatory	6 (2)
Autologous stem cell transplant	3 (1)
Untreated	20 (7)

CKD, chronic kidney disease; CrCl, creatinine clearance; eGFR, estimated glomerular filtration rate; NT‐proBNP, N‐terminal prohormone of brain natriuretic peptide; sFLC, serum free light chains.

Renal and cardiac amyloidosis were present in 208/285 (73%) and 182/285 (64%) of patients respectively; only 14 patients had systemic AL amyloidosis without evidence of either cardiac or renal involvement. The median uRBPCR was 69·4 µg/mmol in patients with renal involvement and 17·6 µg/mmol in patients without renal involvement, 63 of whom had cardiac involvement. The median uRBPCR, uPCR, uACR and percentage with altered FE_PO4_, stratified by chronic kidney disease (CKD) Stage are shown in Table III. The percentage of patients with altered FE_PO4_ increased with advancing CKD Stage, from 11% in CKD Stage 1 to 92% in CKD Stage 5. There was no significant difference in uRBPCR between patients with ALλ and ALκ amyloid (*P* = 0·081).

**Fig 1:**
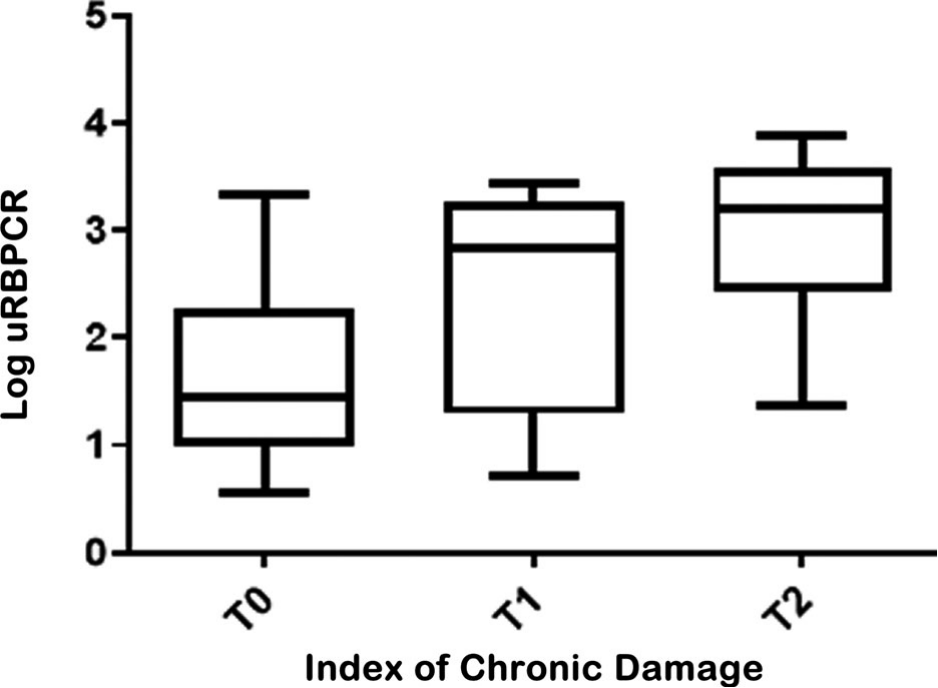
Box plot showing relationship between the urinary retinol binding protein 4/creatinine ratio (uRBPCR) and index of chronic damage on renal histology (*P* < 0·001, Kruskal–Wallis test).

Quantification of Log uRBPCR at baseline correlated with several serum and urine biomarkers, most notably serum creatinine (*r* = 0·618, *P* < 0·0001), creatinine clearance (CrCl) (*r* = 0·613, *P* < 0·0001) and less so uPCR (*r* = 0·422, *P* < 0·0001), as well as fractional excretion of both urate (*r* = 0·563, *P* < 0·0001) and phosphate (*r* = 0·450, *P* < 0·0001). Of note however, among 134 patients with uPCR of <300 mg/mmol, 38 had a uRBPCR of >100 µg/mmol including 12 with a uRBPCR of >1000 µg/mmol. Baseline demographics of patients according to uRBPCR <100 µg/mmol compared to those with uRBPCR ≥100 µg/mmol are listed in Table II. Patients with baseline uRBPCR of ≥100 µg/mmol were older, had significantly higher uPCR and lower eGFR at diagnosis compared to those with baseline uRBPCR of <100 µg/mmol. There was a strong correlation between uRBPCR and index of chronic damage in renal biopsy specimens (*r* = 0·561, *P* < 0·0001) (Figure 1).

**Table II:** Baseline demographics of patients according to urinary retinol binding protein 4/creatinine ratio (uRBPCR) of <100 µg/mmol compared to those with uRBPCR ≥100 µg/mmol.

Demographic or clinical characteristic	uRBPCR <100 µg/mmol *n* = 174	uRBPCR ≥100 µg/mmol *n* = 111	*P*
Age, years, median (range)	67·9 (29·6–93·0)	71·9 (39·5–90·1)	<0·0001
Renal involvement, *n*	110	98	
Cardiac involvement, *n*	117	65	
Serum creatinine, mmol/l, median (range)	83 (34–250)	166 (36–609)	<0·0001
eGFR, ml/min/1·73 m^2^, median (range)	77 (23–100)	33·5 (10–100)	<0·0001
CKD stage, *n*			<0·0001
1	51	5	
2	84	21	
3	27	46	
4	11	27	
5	1	12	
CrCl, ml/min, median (range)	85 (19–274)	39 (4–157)	<0·0001
24‐h urinary protein loss, g, median (range)	1·7 (0·1–33)	4·9 (0·1–42)	<0·0001
Urinary protein creatinine ratio, mg/mmol, median (range)	220 (8–1533)	661 (15–5045)	<0·0001
Urinary albumin creatinine ratio, mg/mmol, median (range)	118 (0·5–952)	207 (0·61–>4000)	0·0171
NT‐proBNP, ng/l, median (range)	1471 (<50–43 774)	2986 (123–204 222)	0·025
Troponin T, ng/l, median (range)	41 (2–326)	60 (9–458)	0·0003
Amyloidogenic light chain, *n*			
Lambda	138	81	
Kappa	36	30	
Bence Jones protein, *n*			0·5999
Present	115	82	
Absent	59	29	
λ sFLC in AL (lambda) amyloid, mg/l, median (range)	178 (13–2685)	173 (13–1483)	0·5085
κ sFLC in AL (kappa) amyloid, mg/l, median (range)	328 (13–3130)	230 (20–2251)	<0·0001
Index of chronic damage on renal histology, *n*			
T0 (mild)	43	22	
T1 (moderate)	4	10	
T2 (severe)	4	21	

CKD, chronic kidney disease; CrCl, creatinine clearance; eGFR, estimated glomerular filtration rate; NT‐proBNP, N‐terminal prohormone of brain natriuretic peptide; sFLC, serum free light chains.

**Table III:** Relationship between baseline glomerular filtration rate stratified by chronic kidney disease (CKD) stage and urinary retinol binding protein 4/creatinine ratio (uRBPCR), urinary protein creatinine ratio (uPCR), urinary albumin/creatinine ratio (uACR) and fractional excretion of phosphate.

CKD Stage	*N*	uRBPCR µg/mmol, median (IQR)	uPCR, mg/mmol, median (IQR)	uACR, mg/mmol, median (IQR)	FE_PO4_, % abnormal
1	56	11·0 (6·6–26·7)	290 (37–586)	140 (3·9–417)	11
2	105	16·9 (7·4–75·7)	322 (35·5–643)	175 (7·4–387)	9
3A	41	44·0 (13·1–503·9)	307 (93–747)	111 (9·6–288)	20
3B	32	449 (24–935)	194 (62–651)	165 (27·1–373)	50
4	38	1418 (477–2055)	385 (58–981)	133 (16·6–398)	58
5	13	4043 (2828–5992)	1003 (579–2183)	491 (106–624)	92

FE_PO4_, fractional excretion of phosphate.

The median follow‐up was 18·7 months from study enrolment. At the time of censoring, 86 patients had died with a median survival from baseline by Kaplan–Meier analysis that was not reached as the survival probability never fell below 65%. A total of 16 patients were on renal replacement therapy (RRT), all of whom had renal involvement at presentation. The median time to ESRD by Kaplan–Meier analysis was not reached but among the 16 patients who were dialysis‐dependent at the time of censoring, the median time from baseline to RRT was 3·9 months.

### Renal survival

#### Result in the whole cohort (*n* = 285)

**Fig 2:**
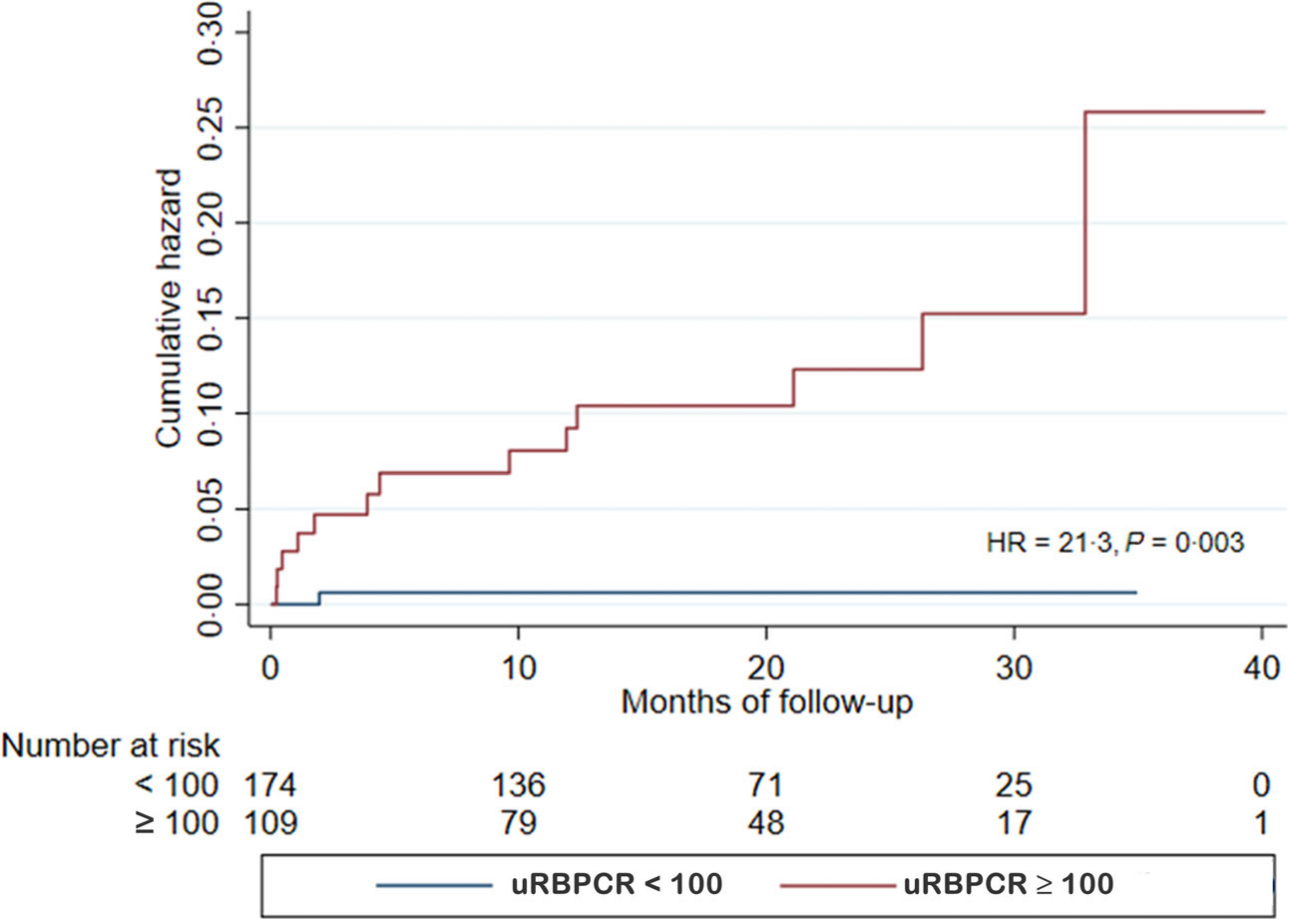
Renal survival by Kaplan–Meier analysis stratified by baseline urinary retinol binding protein 4/creatinine ratio (uRBPCR) of ≥100 µg/mmol or <100 µg/mmol (*P* = 0·003, Log‐rank test).

Univariable analysis demonstrated that baseline Log uRBPCR {hazard ratio [HR] 3·94, [95% confidence interval (CI) 2·02–7·69]; *P* < 0·0001} (Table IV), uPCR [HR 1·10, (95% CI 1·04–1·16); *P* = 0·001] and eGFR [HR 0·62, (95% CI 0·48–0·79); *P* < 0·001] were highly predictive of ESRD in the whole cohort, whilst baseline uACR did not predict ESRD [HR 0·98, (95% CI 0·99–1·01); *P* = 0·822]. Bivariable analysis incorporating uPCR and eGFR demonstrated that eGFR remained predictive of renal survival [HR 0·68, (95% CI 0·53–0·88); *P* = 0·003] but uPCR was not [HR 1·05, (95% CI 0·99–1·12); *P* = 0·075]. However, bivariable analysis incorporating baseline eGFR and Log uRBPCR demonstrated that Log uRBPCR remained highly predictive of renal survival [HR 2·65, (95% CI 1·06–6·64); *P* = 0·038] whilst eGFR was not [HR 0·98, (95% CI 0·95–1·01); *P* = 0·251] (Table V). Finally, when eGFR, uPCR and Log uRBPCR were incorporated into the same multivariable model, none of the variables were significant; this is perhaps not surprising given the correlation between uPCR and Log uRBPCR highlighted above. Kaplan–Meier analysis of renal survival stratified by baseline uRBPCR of ≥ 100 or < 100 µg/mmol showed that uRBPCR of ≥ 100 µg/mmol was highly predictive of ESRD [HR 21·3, (95% CI 2·8–163·2); *P* = 0·003] (Table V; Figure 2).

**Table IV:** Univariable analysis of baseline Log urinary retinol binding protein 4/creatinine ratio (uRBPCR) with outcomes of dialysis and renal progression in; whole cohort, renal involvement, as well as sub analysis based upon baseline estimated glomerular filtration rate (eGFR) and clonal response at 3 months.

Variable	Dialysis	Dialysis	Renal progression 6 months	Renal progression 6 months
Log uRBPCR	Whole cohort	Renal involvement	Whole cohort	Renal involvement
HR (95% CI); *P*:				
All	3·94 (2·02–7·69); <0·0001	2·97 (1·50–5·87); 0·002	1·55 (1·13–2·12); 0·006	1·43 (1 01–2 02); 0·042
eGFR ≥ 30 ml/min/1·73 m^2^	4·11 (1·45–11·65); 0·008	3·42 (1·19–9·85); 0·020	2·01 (1·34–3·01); 0·001	2·06 (1 31–3 23); 0·002
eGFR<30 ml/min/1·73 m^2^	1·30 (0·35–4·82); 0·693	0·81 (0·20–3·19); 0·758	0·96 (0·25–3·69); 0·953	0·75 (0·17–3·34); 0·706
CR/VGPR* at 3 months	3·7 (1·06–12·87); 0·039	2·18 (0·56–8·44); 0·260	1·10 (0·65–1·88); 0·722	0·88 (0·48–1·63); 0·692
PR/NR* at 3 months	6·72 (1·83–24·74); 0·004	5·49 (1·47–20·44); 0·011	2·99 (1·61–5·53); <0·0001	2·76 (1·41–5·39); 0·003

CI, confidence interval; CR, complete response; HR, hazard ratio; NR, non‐responder; PR, partial response; VGPR, very good partial response.

**Table V:** Multivariable analysis of Log urinary retinol binding protein 4/creatinine ratio (uRBPCR), estimated glomerular filtration rate (eGFR) and urinary protein creatinine ratio (uPCR).

Multivariable	Dialysis	
	Whole cohort	
	HR (95% CI)	*P*
Baseline		
eGFR (ml/min/1·73 m^2^)	0·68 (0·53–0·88)	0·003
uPCR, (mg/mmol)	1·05 (0·99–1·12)	0·075
Log uRBPCR (µg/mmol)	2·65 (1·06–6·64)	0·038
eGFR (ml/min/1·73 m^2^)	0·98 (0·95–1·01)	0·251
Log uRBPCR ≥100 µg/mmol vs. Log uRBPCR <100 ug/mmol	21·3 (2·8–163·2)	0·003

Among patients with baseline eGFR of ≥30 ml/min/1·73 m^2^, Log uRBPCR was highly predictive of ESRD [HR 4·11, (95% CI 1·45–11·65); *P* = 0·008] as opposed to patients with a baseline eGFR of <30 ml/min/1·73 m^2^ in whom it was not [HR 1·30, (95% CI 0·35–4·82); *P* = 0·693]. Baseline Log uRBPCR was highly predictive of ESRD in both patients who failed to achieve a haematological CR/VGPR at 3 months [HR 6·72, (95% CI 1·83–24·74); *P* = 0·004] and in those who did achieve a CR/VGPR at this time‐point [HR 3·7, (95% CI 1·06–12·87); *P* = 0·039].

#### Results in renal amyloidosis cohort (*n* = 208) (Table IV)

Univariable analysis demonstrated that baseline Log uRBPCR was highly predictive of ESRD in patients with renal amyloidosis [HR 2·97, (95% CI 1·50–5·87); *P* = 0·002], although on multivariable analysis incorporating baseline eGFR, neither Log uRBPCR [HR 2·12, (95% CI 0·85–5·28); *P* = 0·106] nor baseline eGFR [HR 0·98, (95% CI 0·95–1·01); *P* = 0·312] predicted ESRD. However, among patients with renal involvement and baseline eGFR ≥ 30 ml/min/1·73 m^2^, Log uRBPCR was predictive of ESRD [HR 3·42, (95% CI 1·19–9·85); *P* = 0·020] whilst it was not among those with baseline eGFR <30 ml/min/1·73 m^2^ [HR 0·81, (95% CI 0·20–3·19); *P* = 0·758]. Baseline Log uRBPCR was particularly predictive of ESRD in patients with renal amyloidosis who failed to achieve a haematological CR/VGPR with chemotherapy at 3 months [HR 5·49, (95% CI 1·47–20·44); *P* = 0·011] but did not reach statistical significance among those who achieved a CR/VGPR with chemotherapy at this time‐point [HR 2·18, (95% CI 0·56–8·44); *P* = 0·260].

### Renal progression

#### Results in whole cohort (*n* = 285) (Table IV)

Renal progression occurred in 69 patients at 6 months and 61 patients at 12 months (fall in numbers over time due to mortality). Univariable analysis demonstrated that baseline Log uRBPCR was predictive of renal progression both at 6 months [HR 1·55, (95% CI 1·13–2·12); *P* = 0·006] and at 12 months [HR 1·56, (95% CI 1·10–2·22); *P* = 0·013]. Multivariable analysis, incorporating baseline eGFR, demonstrated that baseline Log uRBPCR was predictive of renal progression at 6 months [HR 1·80, (95% CI 1·16–2·82); *P* = 0·009] and at 12 months [HR 1·91, (95% CI 1·18–3·07); *P* = 0·008]. Among patients with baseline eGFR ≥30 ml/min/1·73 m^2^, baseline Log uRBPCR was highly predictive of renal progression at both 6 [HR 2·01, (95% CI 1·34–3·01); *P* = 0·001] and 12 months [HR 1·81, (95% CI 1·17–2·81); *P* = 0·008], although this was not the case in patients with baseline eGFR <30 ml/min/1·73 m^2^ at either 6 [HR 0·96, (95% CI 0·25–3·69); *P* = 0·953] or 12 months [HR 1·05, (95% CI 0·26–4·25); *P* = 0·948]. Among patients who failed to achieve a haematological CR/VGPR with chemotherapy at 3 months, baseline Log uRBPCR was highly predictive of renal progression at both 6 months [HR 2·99, (95% CI 1·61–5·53); *P* < 0·0001] and 12 months [HR 1·94, (95% CI 1·15–3·25); *P* = 0·012] but this was not so among those who did achieve a CR/VGPR at 3 months with regards to renal progression at 6 [HR 1·10, (95% CI 0·65–1·88); *P* = 0·722] and 12 months [HR 1·12, (95% CI 0·71–2·03); *P* = 0·498]. Similarly, baseline Log uRBPCR predicted renal progression at 12 months among patients who failed to achieve a CR/VGPR at 6 months [HR 1·94, (95% CI 1·08–3·50); *P* = 0·027] but not in those who did achieve CR/VGPR at 6 months.

#### Results in renal amyloidosis cohort (*n* = 208) (Table IV)

Univariable analysis demonstrated that among patients with renal amyloidosis, baseline Log uRBPCR was predictive of renal progression at 6 months [HR 1·43, (95% CI 1·01–2·02); *P* = 0·042] but not at 12 months [HR 1·26, (95% CI 0·86–1·84); *P* = 0·237]. Multivariable analysis, incorporating baseline eGFR, demonstrated that among patients with renal amyloidosis, baseline Log uRBPCR was predictive of renal progression at 6 months [HR 1·74, (95% CI 1·07–2·82); *P* = 0·025], and particularly so in those with baseline eGFR ≥30 ml/min/1·73 m^2^ [HR 2·06, (95% CI 1·31–3·23); *P* = 0·002]. In patients with renal amyloidosis who failed to achieve a CR/VGPR with chemotherapy at 3 months, baseline Log uRBPCR was highly predictive of renal progression at 6 months [HR 2·76, (95% CI 1·41–5·39); *P* = 0·003] but this was not so among those who did achieve a CR/VGPR at 3 months [HR 0·88, (95% CI 0·48–1·63); *P* = 0·692].

### Patient survival

There was no significant association between Log uRBPCR and overall patient survival [HR 0·96, (95% CI 0·77–1·20); *P* = 0·731].

## Discussion

Renal involvement in systemic AL amyloidosis is common and risk stratification of patients is currently based upon eGFR and proteinuria. In the present study, we show for the first time in a large prospective study of newly diagnosed patients with AL amyloidosis that uRBPCR at baseline is a strong independent predictor of both renal progression and ESRD.

Despite the long‐known association between monoclonal gammopathies and PTD, we demonstrate in the present study the specific association between presence of renal amyloid and quantification of uRBPCR. In addition, we show the correlation between uRBPCR and both uPCR and GFR, as well as other measures of PTD such as fractional excretion of urate and phosphate. Indeed, increased fractional excretion of both phosphate and urate was present in 20% of patients with CKD Stage 3a, increasing to ~92% of those with CKD Stage 5. uRBPCR was not associated with uACR, and despite its association with uPCR, 28% of patients with sub‐nephrotic proteinuria had markedly elevated uRBPCR and 13% (14/106) of patients with proteinuria of >5 g/24 h had uRBPCR in the normal range. Similarly, uRBPCR was sometimes markedly elevated in patients with preserved GFR.

Our present study demonstrated that whilst baseline uRBPCR was predictive of both renal progression and dialysis in the whole cohort, independently of GFR and clonal response to chemotherapy, it appeared to be most predictive of these hard outcome measures among patients with a presenting eGFR of ≥30 ml/min/1·73 m^2^. This is exactly the group of patients with systemic AL amyloidosis in whom one would hope to salvage renal function and avoid dialysis, previously deemed ‘low risk’ of developing ESRD. However, we show in the present study that among patients with an eGFR of ≥30 ml/min/1·73 m^2^ at baseline, each incremental increase in Log uRBPCR increases the risk of renal progression twofold and the risk of dialysis more than fourfold. This finding, coupled with the correlation between uRBPCR and ‘Index of Chronic damage’ on renal histology, offers new insight into the importance of PTD in renal AL amyloidosis, hitherto neglected in comparison to glomerular dysfunction.

Clonal response to chemotherapy is a well‐recognised predictor of overall survival, renal survival and renal response in AL amyloidosis. Baseline uRBPCR was predictive of both renal progression and ESRD in patients who achieved a CR/VGPR with chemotherapy but was a particularly strong predictor of these outcome measures among patients who failed to achieve this degree of haematological response. This is entirely plausible from a pathophysiological perspective; whilst haematological response is the main determinant of ongoing amyloid deposition and therefore disease progression, uRBPCR is likely to represent the effect, either through toxicity or physical presence, of existing AL amyloid deposits on proximal tubular cell function. Most importantly from a clinical perspective, measurement of uRBPCR enables further risk stratification of patients and particularly those with a relatively preserved presenting GFR. Despite the strength of these data, assessment of PTD in AL amyloidosis has its challenges, particularly as uRBP is both filtered at the glomerulus and reabsorbed at the proximal tubule; both compartments of the kidney may be involved to varying degrees in patients with renal AL amyloidosis. Interpretation of elevated uRBP levels may be particularly challenging in patients with advanced CKD at diagnosis who may have both glomerular and tubular dysfunction and we would encourage further validation of our present findings, particularly in this patient group.

Limitations of our present study include the relatively low incidence of dialysis in this cohort of patients. A total of 16 patients required dialysis of whom two were on RRT at presentation. The need for RRT is a key renal end‐point and whilst >70% of patients had evidence of renal involvement at presentation, the relatively low incidence of dialysis dependence in this cohort may also reflect improved chemotherapy options in AL amyloidosis.

In conclusion, the present prospective study of patients with newly diagnosed systemic AL amyloidosis, demonstrates for the first time the role of uRBPCR as a prognostic biomarker for renal progression, which may aid clinicians to risk stratify patients receiving cytotoxic chemotherapy including those previously deemed ‘low risk’ of renal progression and ESRD. Our present data highlights the need for further study of proximal tubular function in other monoclonal gammopathies of renal significance (MGRS), as well as other so called primary glomerulopathies.

## Conflict of Interest

The authors declare no conflict of interest.

## Funding information

This work was funded by the UK Department of Health and facilitated by testing services by the South West Thames Institute for Renal Research with support from BBI Solutions. In addition, this research was funded in part by the Wellcome Trust [110182/Z/15/Z]. For the purpose of open access, the author has applied a CC BY public copyright licence to any Author Accepted Manuscript version arising from this submission.
